# Grand Challenges for Personality and Social Psychology: Moving beyond the Replication Crisis

**DOI:** 10.3389/fpsyg.2017.02068

**Published:** 2017-11-28

**Authors:** Anat Bardi, Marcel Zentner

**Affiliations:** ^1^Department of Psychology, Royal Holloway, University of London, London, United Kingdom; ^2^Department of Psychology, University of Innsbruck, Innsbruck, Austria

**Keywords:** replication crisis, sampling, longitudinal methods, person vs. variable-centered analyses

The replication crisis has shaken the field of personality and social psychology in the past few years, after finding out that well-known effects that we have taken for granted failed to replicate (see, e.g., Marsman et al., 2017). This has happened partly due to reliance on flawed research conduct, such as confusing exploratory and confirmatory statistical tests (e.g., Wagenmakers et al., 2012). But such conduct was not created in a vacuum. It has arisen under pressure to show surprising, mesmerizing findings as a requirement for publication in highly selective journals in the quest for career survival and progression. Much has been written about solutions that would ensure that future research is replicable and reproducible (e.g., Fraley and Vazire, 2014; Nosek et al., 2015; Simonsohn, 2015), including in our journal (e.g., Everett and Earp, 2015) and in the current Research Topic on “Grand Challenges in Psychology” (Stevens, 2017). Things are still changing rapidly, with recent solutions already being questioned (e.g., pre-registration practices, see Wicherts and Veldkamp, 2017). We therefore do not wish to re-iterate what has been written so eloquently by others (e.g., Stevens, 2017). Instead, we only wish to highlight a few ways in which publishing in Frontiers can help overcome this crisis and we then move on to discuss research avenues that we believe would particularly enhance knowledge and understanding in personality and social psychology.

In personality and social psychology effect sizes tend to be small to moderate, in part because social behavior is complex and influenced by many factors. As a consequence, what any one study can reveal is at best a small fraction of these factors. Hence, our findings tend to be sensitive to the particular statistical analysis conducted, the particular population sampled, and the particular social context. Instead of seeing this as a problem, we encourage recognizing this variability, and publishing null results, which is possible in Frontiers as long as there is sufficient statistical power and the design is valid. We encourage transparent reporting of the ways in which the analyses were conducted and we also strongly recommend including statistical indicators that are not based on significance testing, such as effect sizes. As opposed to significance testing, effect size estimates provide direct information about the strength of an effect, they allow for comparisons across studies independently of sample sizes or type of test, and seem also better suited to the complexity of human social behavior than the yes/no answers that significance testing provides.

In Frontiers, findings do not have to be striking or unprecedented to merit publication. Instead, it is sufficient to report valid research. It is then up to the research community and the wider public to judge the importance of the research, and potentially build on it and develop the research question further. The possibility to publish null results means that the public and research community are routinely informed about known effects that have not been replicated. Lack of replication should not be seen as a failure. Instead, it can help us understand better under which circumstances an effect exists, as the replication may be done under different circumstances, languages, social contexts, cultures, and populations.

All the solutions that have been proposed so far by the research community cannot operate in a vacuum. As long as reinforcement strategies for career survival and progression require publications of surprising or attention-grabbing findings, researchers will remain under pressure to do what they can to produce such findings. We call other journals to re-think the importance of surprises and instead emphasize rigorous research, whether it produces surprising findings or not.

Although the replication crisis is usually seen as arising from methodological flaws, it is also a symptom of structural issues in today's personality and social psychology that end up undermining the reliability of research findings. By structural issues we mean trends, conventions and practices in the production and appraisal of scientific knowledge that are upheld by institutions. In what follows, we focus on scope, range, and person-centeredness as aspects that are critical to reproducibility and validity, but are increasingly compromised by structural problems in today's social psychological and personality science.

## Scope

A few decades ago, Gleitman (1983) characterized psychology as “a loosely federated intellectual empire that stretches from the domains of the biological sciences on one border to those of the social sciences on the other” (p. 504). Today, this statement could be applied just as well to the domain of personality and social psychology. In a field that has grown so extraordinarily vibrant and diverse, integration would seem more important than ever. Yet, developing a unified theory of the universe has turned out to be a more attainable goal than developing a unified theory of social behavior. As a result, many theories in personality and social psychology are essentially mini theories, intended to explain particular forms of behavior under a set of circumstances. Although theories with a broader, more inclusive scope exist (e.g., Block, 2002; Mischel, 2004), the picture of a fragmented science, consisting of many parallel mini theories and isolated effects, prevails. Rather than repeating what others have said on the subject of scope and integration (e.g., Caccioppo, 2007; Cleeremans, 2010), we would like to point out some contingencies between scope and likelihood of replication.

At first sight, research approaches with a narrow focus may seem more likely to yield reproducible findings than those that aim at revealing general principles of social and personality processes. However, discounting much of the complex and multi-determined nature of social behaviors can also make findings more brittle. For example, Wheeler and DeMarree (2009) identified 21 moderators of priming effects. When many potentially relevant factors are left out of a study, for reasons of parsimony or control, they do not therefore cease to influence the behavior of interest. Rather, their influence becomes imponderable—it can be negligible in one study, but more prominent in another. The inconsistent influence of factors that are left out of studies is bound to result in inconsistent findings.

In contrast, work that succeeds in integrating the complexity of factors involved in social behavior and personality may be less vulnerable to replication failures because a large number of otherwise unpredictable variables is factored in. Complexity may be accounted for by adopting one of two basic strategies. One is to include the largest number of potentially significant extraneous variables and treat them as control variables. Because measuring many extraneous variables is arduous, this strategy tends to be neglected. Another, yet more taxing strategy, is to directly model the effects of potentially significant extraneous variables by drawing from theory or empirical evidence.

To engage in extensive work of this kind, one needs to be prepared to work for the long haul and often outside beaten tracks. However, such work is at odds with current reinforcement strategies for career progression that urge and reward rapid rates of publication. As long as this is the case, there is minimal incentive to engage in work with a large and inclusive scope. We call on peers and colleagues to raise awareness for the limitations current career advancement practices impose on the scope of social psychological and personality science, and to think about ideas for changing current practices.

## Range

Another key component of research in personality and social psychology is range. Range relates to the type of people to which any given finding or mechanism may apply. In response to pressures for rapid rates of publication, social and personality psychologists often use samples that are easy to collect and acquire—typically, student or other convenience samples. Unfortunately, this practice significantly restricts the range to which any finding may be applied. The likelihood of replication of an effect is thereby reduced because it does not take much to stray from the narrow range for which the effect has been originally established. In support of this point, a meta-analysis by Peterson (2001) found that effects in domains such as gender differences, persuasion, or aggression can differ substantially depending on whether student samples were used or not. As editors, we strongly encourage studies that include samples with a wide range of ages, ethnicities, income-levels and professions. Including socio-demographically diverse samples will not only widen our understanding of social and personality processes, it will also help to identify findings that are resistant against variations in sample composition and thus more likely to replicate.

While samples of substantial diversity cannot always be recruited, it is often possible to estimate how much any given sample deviates from the general population or from the populations to which one wishes to generalize. National bureaus of statistics hold information on regional and national populations, including age, education, profession, income, or health. Some databases also include information on values, attitudes and personality. Analyzing how a given research sample relates to the relevant population norms has the benefit of leaving readers with a better sense of what can be realistically concluded from the findings.

Just as important as our knowledge about generalizability across samples is our knowledge about what happens within samples over time. Finding relationships between social psychological and personality attributes across time is crucial for several reasons. One is the higher reliability of measures that are taken repeatedly over time (Zentner et al., 2014). A second is directionality. In non-experimental designs, direction of effects cannot be inferred when all variables are measured simultaneously (as in cross-sectional designs), and at the very least the presumed cause and effect should be measured at different points in time (Duckworth et al., 2010). A third reason lies in the comparatively patchy knowledge we have of social and personality development from birth to adulthood. What is presently known about the development of key attributes such as aggression, anxiety, executive functioning, or attachment security from infancy to adulthood rests on less than a dozen prospective studies (Zentner and Shiner, 2015). As a result, the gaps in the available empirical evidence about the role of early childhood precursors of personality and psychopathology must be filled with speculations. To establish a reliable body of findings on personality development, long-term longitudinal research needs to be embraced more pervasively. Unfortunately, the incentive for longitudinal exploration is curbed by the same structural factors that compromise the scope of research in personality and social psychology. We call on institutions to create conditions that incentivize and reward longitudinal research.

## Person-centerdness

Finally, we would like personality and social psychology to develop and use research paradigms that provide knowledge about persons. Although this point may seem obvious, most current research in personality and social psychology studies variables rather than persons. Decomposing individuals' minds into sets of personality traits or variables is a powerful tool of abstraction and formalization. In the end, however, our discipline is about the person as a whole. Person-centered approaches, rather than studying personality traits in isolation, focus on the total constellation of traits that define each person, and the way these traits work together as a dynamic, integrated system.

Although person-centered research made a modest comeback in recent years, it still faces headwinds due to its relative unfamiliarity and unwarranted association with case study methodology. Yet, person-centered approaches offer ways of abstraction that are similarly powerful to those offered by variable-centered approaches (e.g., cluster analysis, latent class and profile analysis, prototype matching), all while taking the pattern of dispositions within persons into account (e.g., Chapman and Goldberg, 2011; Asendorpf, 2015; Borg et al., 2017). As shown in a collection of contributions recently published in our journal (Beckmann and Wood, 2017), person-centered approaches also offer distinct benefits to the study of within-person variation over time. Finally, findings about persons, or types of persons, are more intuitive to grasp for non-experts than are findings about variables, or relationships between variables.

For example, McCartney and Rosenthal (2000) pointed out that policymakers cannot grasp the magnitude of effects as they are reported in many research reports. One way to make these effects more accessible is by recasting the associations in terms of success rates for groups of individuals (e.g., those exhibiting a certain characteristic or receiving a given treatment). Because person-centered research already presents its findings in terms of types of individuals, it offers some of the interpretational advantages highlighted by McCartney and Rosenthal. For purposes of illustration, compare the following two statements: (a) Undercontrolled children fell behind resilient children by a year of schooling over the course of elementary school on average. (b) The effect size between children's conscientiousness and neuroticism and the rate of academic decline over the course of elementary school years was *r* = −.xx and *r* = +.yy, respectively (see Hart et al., 2003). By highlighting the benefits of person-centered approaches, we are not questioning the value of traditional dimensional approaches. What we hope to see is a better balance between both approaches.

In principle, social and personality psychologists should be obvious experts to turn to when some everyday event requires expertise in personality or social psychology. But we rarely see them on BBC or CNN. While we do not advocate presence in the media for its own sake, such presence can help inform audiences, including policymakers, about key issues studied by personality and social psychologists, from mechanisms underlying racial discrimination to risk factors for divorce. By attaining a better balance between variable- and person-centered approaches to personality and social behavior, we will not only gain knowledge that can be more readily applied to psychological understanding of the person, we might also see our field's outreach enhanced.

## Author contributions

All authors listed have made a substantial, direct and intellectual contribution to the work, and approved it for publication.

### Conflict of interest statement

The authors declare that the research was conducted in the absence of any commercial or financial relationships that could be construed as a potential conflict of interest.
